# Possible species discrimination of a blotched nerite *Nerita albicilla* with their distribution pattern and demographic history in the Indo-Pacific

**DOI:** 10.1038/s41598-023-31004-0

**Published:** 2023-03-20

**Authors:** Seonghyeon Hong, Bia Park, Gyeongmin Kim, Eun Hwa Choi, Ui Wook Hwang

**Affiliations:** 1 Department of Biology, Teachers College and Institute for Phylogenomics and Evolution, Kyungpook National University, Daegu, 41566 Republic of Korea; 2 School of Life Sciences, Graduate School, Kyungpook National University, Daegu, 41566 Republic of Korea; 3 Phylomics Inc., Daegu, 41910 Republic of Korea; 4 Institute for Korean Herb-Bio Convergence Promotion, Kyungpook National University, Daegu, 41566 Republic of Korea; 5 School of Industrial Technology Advances, Kyungpook National University, Daegu, 41566 Republic of Korea

**Keywords:** Phylogenetics, Population genetics

## Abstract

The blotched nerite *Nerita albicilla* (Linnaeus 1758) is distributed in intertidal areas of the Indo-Pacific. In South Korea, it has been found only in the southernmost region of Jeju Island so far. Owing to its limited distribution, it can be a promising intertidal species helpful for monitoring global climate change effects in the Korean Peninsula. We performed population genetic analyses based on 393 *COI* haplotypes from 697 *N. albicilla*, including 167 from this study and 530 from public databases. The results showed that there are two distinct genetic lineages in *N. albicilla*: PAIO (Palearctic, Australasia, Indo-Malay, and Oceania) and Afrotropic lineages. DNA barcoding gap analyses indicated that the two lineages could be differentiated into two different species: *N. albicilla* (PAIO) and *N. originalis* sp. nov. (Afrotropic) (3.96%). Additionally, it was revealed that their divergence time was ca. 5.96 Ma and dramatic diversification of *COI* haplotypes occurred during the late Pliocene and Pleistocene. The results of MDA, BSP, and neutrality test implied recent population size expansion, which was estimated to be ca. 250 Ka. Finally, we discussed whether the observation of *N. originalis* sp. nov. in South Korea is due to the northward migration through ocean currents caused by global warming or due to artificial activity through marine transportation.

## Introduction

The blotched nerite, *Nerita albicilla*, an intertidal marine species, has long-lived planktotrophic larvae^1^, which can be characterized by its depressed spire, sculptured with broad, low spiral ribs, and an outer lip with one or two strong teeth posteriorly and sometimes anteriorly located^2^. Shells commonly reach 24 mm in height and primarily inhabit rubble and cobble fields with low wave energy, and are often associated with inner-reef flats^3,4^. *Nerita albicilla* is widely distributed extending to the shores of East Africa in the west and reaching its eastern boundary at the Cook Islands^5^. However, in South Korea, *N. albicilla* is restricted to the southernmost area of Jeju Island, located approximately 90 km south of the mainland^6^. Due to the interesting distribution pattern of *N. albicilla*, the National Institute for Biological Resources, Ministry of Environment, South Korea was designated as one of the 100 Climate Change Biological Indicator Species (CBIS) in 2010 for continuously monitoring and predicting climate change caused by global warming^7^.

The evolutionary rate of mitochondrial DNA is faster than that of nuclear DNA, enough to examine genetic diversity and differentiation of not only closely related sibling species but also phylogeographically diverged genetic groups within a single species^8–12^. These characteristics make mitochondrial DNA particularly appropriate for tracing recent evolutionary history, including colonization or translocation events and population bottlenecks^13^. In recent decades, many complete mitochondrial genomes mollusc consisting of 13 protein-coding genes, 22 tRNA genes, 2 rRNA genes, and one or two control region(s) have been sequenced and characterized^14–16^. Especially, a single mitochondrial gene, such as the cytochrome *c* oxidase subunit I gene (*COI*), can be used as an effective biological species identification system^9,17^ and can be informative in tracing the movement of a species^18^. This marker can also genetically distinguish closely related marine invertebrate species^12^, despite its short sequence (~ 543 bp). Crandall et al.^5^ analyzed the demographic history and phylogeographical patterns of *N. albicilla* and *N. plicata*. Using 529 *COI* sequences of *N. albicilla*, they found that *N. albicilla* exhibited a reciprocal monophyly of Indian and Pacific Ocean populations, indicating that *N. albicilla* comprises two distinct genetic lineages: clades A and B*.* Since then, no population genetic study of *N. albicilla* in the Indo-Pacific has been conducted.

In the present study, we attempted to characterize the genetic diversity and population genetic structure of Indo-Pacific *N. albicilla* based on 393 *COI* haplotypes extracted from 697 individuals, consisting of 530 individuals retrieved from public databases and 167 newly collected from five different sites located in the southernmost areas of Jeju Island, South Korea. The results confirmed that the presence of two distinct genetic lineages in Indo-Pacific *N. albicilla*, called Afrotropic and PAIO (Palearctic, Australasia, Indo-Malay, and Oceania) lineages, indicating probable *N. albicilla* species discrimination. In addition, we analyzed the DNA barcoding gap and its distribution patterns, which showed that the two genetic lineages may be reclassified into two independent *Nerita* species: *N. albicilla* (PAIO) and *N. originalis* sp. nov. (Afrotropic). In addition, their divergence times and demographic histories were explored. It is expected that the present results will be helpful for monitoring distribution patterns and population expansion of intertidal marine gastropods, as well as for understanding mechanisms of species discrimination of marine gastropods.

## Results

### Genetic diversity of *N. albicilla* based on *COI*

**Figure 1:**
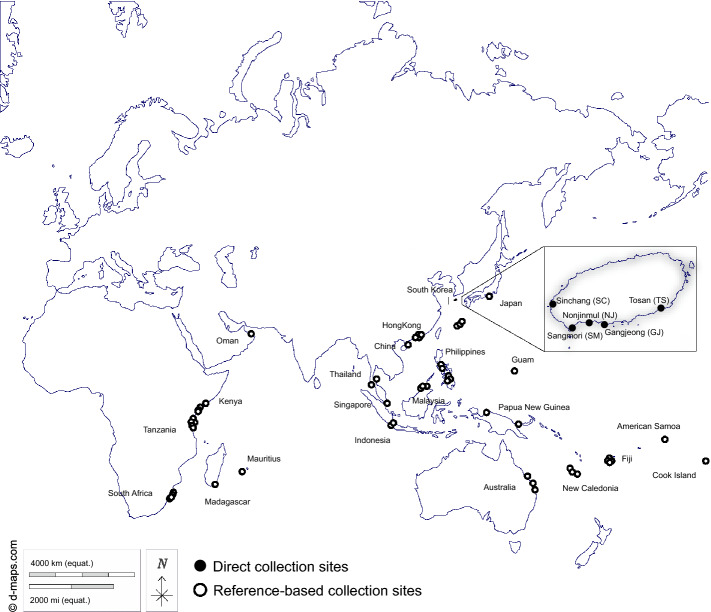
A map showing the collection sites of *Nerita albicilla* inhabiting intertidal areas of the Indo-Pacific including the Korean Peninsula (N = 697). Black dots represent the sites where the samples are directly collected in this study and white dots with black border are the collection sites where the samples are from references. Two individuals of Afrotropic in Jeju Island are directly collected from GJ and SM. The maps are provided from the d-maps site (https://d-maps.com) and the figure was edited by Adobe Illustrator v.22.2 (https://www.adobe.com).

A total of 167 *N. albicilla* individuals were collected from five intertidal regions on Jeju Island, South Korea (Fig. 1; Table 1). From the collected samples, we amplified the *COI* barcoding region using PCR, and the resultant 575-bp PCR products were sequenced. In addition, we retrieved 530 previously published *COI* sequences of *N. albicilla*: 524 from Eric D. Crandall from https://geome-db.org/query and six from the NCBI GenBank database. As a result, a total of 393 *COI* haplotypes sequences (Data S1) were defined from 697 *N. albicilla* individuals which were collected from 26 localities: five from Jeju Island in South Korea and 21 from other locations including the Palearctic (Japan and Oman), Indo-Malay (China, China-Hong Kong, Indonesia, Japan-Okinawa, Malaysia, Philippines, Singapore, and Thailand), Australasia (Australia, New Caledonia, and Papua New Guinea), Oceania (American Samoa, Cook Island, Fiji, and Guam), and Afrotropic region (Kenya, Madagascar, Mauritius, South Africa, and Tanzania).

**Table 1:** List of collection sites and the number of *Nerita albicilla* individuals in the Indo-Pacific.

Nations (Populations)		Collection sites	N	Biogeographic realm	References
South Korea	(GJ)	4968–7, Gangjeong-dong, Seogwipo-si, Jeju-do	60	Palearctic	This study
	(NJ)	253, Yeraehaean-ro, Seogwipo-si, Jeju-do	10	Palearctic	This study
	(SC)	1446–3, Sinchang-ri, Hangyeong-myeon, Jeju-si, Jeju-do	30	Palearctic	This study
	(TS)	30–3, Tosan-ri, Pyoseon-myeon, Seogwipo-si, Jeju-do	30	Palearctic	This study
	(SM)	87–27, Sangmo-ri, Daejeong-eup, Seogwipo-si, Jeju-do	37	Palearctic	This study
American Samoa (AMS)		Onenoa	8	Oceania	Crandall et al. (2008)
Australia (QSLD)		Horseshoe Bay, Bowen	10	Australasia	Crandall et al. (2008)
		Redcliffe, Moreton Bay	11	Australasia	Crandall et al. (2008)
		Elliott Heads, Bundaberg	9	Australasia	Crandall et al. (2008)
China (CH)		Haikou, Hainan province	1	Indo-Malay	Feng et al. (2019)
China (HK)		Wu Kai Sha	9	Indo-Malay	Crandall et al. (2008)
		Siulam	1	Indo-Malay	Crandall et al. (2008)
		Yung Shue Long	10	Indo-Malay	Crandall et al. (2008)
		Shek O	10	Indo-Malay	Crandall et al. (2008)
Cook Islands (RAR)		Avarua	30	Oceania	Crandall et al. (2008)
Fiji (FJ)		Uciwai	10	Oceania	Crandall et al. (2008)
		Korotogo	10	Oceania	Crandall et al. (2008)
		Suva	10	Oceania	Crandall et al. (2008)
		Navolau	10	Oceania	Crandall et al. (2008)
Indonesia (INDO)		Yapen -West tip of Serui	19	Indo-Malay	Crandall et al. (2008)
		Alam Kotok	19	Indo-Malay	Crandall et al. (2008)
		Sangiang	18	Indo-Malay	Crandall et al. (2008)
Japan (JP)		Okinawa I., Okinawa Pref	1	Indo-Malay	Williams et al. (2006)
		Shioya -wan	11	Palearctic	Crandall et al. (2008)
		Cape Hedo	9	Palearctic	Crandall et al. (2008)
		Oku	1	Palearctic	Crandall et al. (2008)
		Misaki	20	Palearctic	Crandall et al. (2008)
Kenya (KEN)		Mombasa	9	Afrotropic	Crandall et al. (2008)
		Diani	10	Afrotropic	Crandall et al. (2008)
		Malindi	10	Afrotropic	Crandall et al. (2008)
		Kilifi	10	Afrotropic	Crandall et al. (2008)
Madagascar (MAD)		Taolagnaro	2	Afrotropic	Crandall et al. (2008)
Malaysia (MAL)		Cape Gaya	9	Indo-Malay	Crandall et al. (2008)
		Bak Bak	10	Indo-Malay	Crandall et al. (2008)
		Pantai Kulambu	10	Indo-Malay	Crandall et al. (2008)
Mauritius (MAU)		Souillac	9	Afrotropic	Crandall et al. (2008)
New Caledonia (NC)		Kanumera, Ile de Pins	11	Australasia	Crandall et al. (2008) Frey et al. (2006)
		Roche Percee	10	Australasia	Crandall et al. (2008)
		Poindimie	10	Australasia	Crandall et al. (2008)
Oman (OMAN)		Cemetery Bay Muscat	10	Palearctic	Crandall et al. (2008)
Philippines (PH)		Tubod Mar Bohol	6	Indo-Malay	Crandall et al. (2008)
		Balite Mindoro	4	Indo-Malay	Crandall et al. (2008)
		Sabang Mindoro	10	Indo-Malay	Crandall et al. (2008)
		Doljo Bohol	6	Indo-Malay	Crandall et al*.* (2008)
		Alona Beach Bohol	6	Indo-Malay	Crandall et al. (2008)
Papua New Guinea (PNG)		Huon Peninsula	10	Australasia	Crandall et al. (2008)
South Africa (SAF)		Hibberdene	3	Afrotropic	Crandall et al. (2008)
		Umdloti	7	Afrotropic	Crandall et al. (2008)
		Rocky Bay	11	Afrotropic	Crandall et al. (2008) Frey et al. (2006)
		Mission Rocks	10	Afrotropic	Crandall et al. (2008)
Singapore (SG)		East Coast Pk	10	Indo-Malay	Crandall et al. (2008)
Tanzania (TAN)		Slipway	10	Afrotropic	Crandall et al. (2008)
		Oyster Bay	10	Afrotropic	Crandall et al (2008)
		Msimbazi	1	Afrotropic	Crandall et al*.* (2008)
		Mkwajuni	10	Afrotropic	Crandall et al. (2008)
		Bawe Island	9	Afrotropic	Crandall et al. (2008)
		Kizimkazi	9	Afrotropic	Crandall et al. (2008)
		Matemwe	6	Afrotropic	Crandall et al. (2008) Frey et al. (2006)
		Kigombe	10	Afrotropic	Crandall et al. (2008)
Thailand (TH)		Laem Phanwa Phuket	10	Indo-Malay	Crandall et al. (2008)
		Ao Nang	1	Indo-Malay	Crandall et al. (2008)
		Hat Chaweng	9	Indo-Malay	Crandall et al. (2008)
USA (GUAM)		Anigua	5	Oceania	Crandall et al. (2008)
Total			697		

* ‘N’ indicates the number of individuals.

The genetic diversity estimated with 393 *COI* haplotypes revealed that the average haplotype diversity (h) and nucleotide diversity (π) were 0.989 and 0.02001, respectively (Table 2). The highest haplotype diversity (h = 1) was observed in Nonjinmul (NJ), American Samoa (AMS), Guam (GUAM), Madagascar (MAD), and Singapore (SG), whereas the highest nucleotide diversity (π = 0.02490) was found in Thailand (TH). The most abundant haplotype was NAH37, which was found in 45 *COI* sequences from 697 *N. albicilla* individuals.

**Table 2:** Summary of the population genetic analyses and neutrality tests performed with 697 *COI* sequences from *Nerita albicilla* along the 26 populations.

Populations	N	N_h_	*h*	π	S	k	Tajima’s *D*	Fu’s *F*
GJ	60	47	0.987	0.00884	69	5.08079	− 2.25086**	− 25.52098**
NJ	10	10	1.000	0.00757	18	4.35556	− 1.47275	− 6.51053*
SC	30	26	0.989	0.00783	34	4.50115	− 1.73370*	− 24.12741**
TS	30	25	0.982	0.00671	32	3.85747	− 1.89671*	− 23.88335**
SM	37	30	0.983	0.00864	55	4.96547	− 2.25645**	− 25.21028**
AMS	8	8	1.000	0.00932	17	5.35714	− 0.94356	− 3.71314**
CH	1	1	−	−	−	−	−	−
FJ	40	29	0.977	0.00734	39	4.22308	− 1.89318*	− 24.43354**
GUAM	5	5	1.000	0.00800	11	4.60000	− 0.92693	− 1.48053
HK	30	22	0.977	0.00623	28	3.58161	− 1.77275*	− 17.52939**
INDO	56	44	0.986	0.01184	63	6.80714	− 1.73392*	− 25.09229**
JP	41	34	0.990	0.00871	34	5.00854	− 1.87188*	− 25.53299**
KEN	39	33	0.991	0.00807	33	4.63833	− 2.07667**	− 25.64423**
MAD	2	2	1.000	0.00870	5	5.00000	−	−
MAL	29	21	0.929	0.00598	29	3.43842	− 1.93783*	− 16.44266**
MAU	9	8	0.972	0.00705	14	4.05556	− 1.02086	− 3.33990*
NC	31	25	0.985	0.01089	52	6.26237	− 1.92719*	− 15.88368**
OMAN	10	8	0.956	0.00734	12	4.22222	− 0.02088	− 2.46767*
PH	32	30	0.994	0.00910	44	5.23185	− 1.90904*	− 25.47418**
PNG	10	9	0.978	0.00672	14	3.86667	− 1.00148	− 4.50734*
QSLD	30	25	0.986	0.00718	33	4.13103	− 1.83399*	− 22.87074**
RAR	30	21	0.954	0.00886	45	5.09655	− 2.04415**	− 11.17394**
SAF	31	27	0.989	0.01015	53	5.83656	− 2.08196**	− 21.78603**
SG	10	10	1.000	0.01360	31	7.82222	− 1.37603	− 4.24424*
TAN	65	48	0.985	0.00729	54	4.19327	− 2.11758**	− 25.84387**
TH	20	19	0.995	0.02490	46	14.31579	0.41746	− 6.90050**
Total	697	393	0.989	0.02001	186	11.5076	− 1.63021*	− 23.62584**

Diversity parameters are given for each locality: N = the number of *COI* sequences (individuals), N_h_ = the number of haplotypes, *h* = haplotype diversity, π = Jukes-Cantor corrected estimates of nucleotide diversity, S = the number of segregation sites, k = the average number of pairwise nucleotide differences, and ‘nd’ = not determined. Statistically significant values are indicated with asterisk: *P < 0.05, **P < 0.01. The localities of the populations are shown in Table 1 and Fig. 1.

### Population genetic analyses

**Figure 2:**
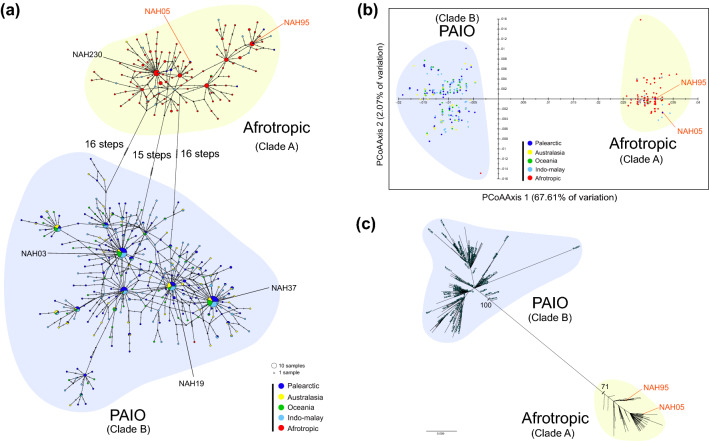
A TCS haplotype network, PCoA analyses, and a unrooted maximum likelihood tree based on the 393 *COI* haplotypes of *Nerita albicilla* in the Indo-Pacific. (**a**) A unrooted TCS haplotype network. Haplotype frequency is related to the size of the circle. Different colors within the nodes refer to different sampling sites divided into the five geographical regions depicted by the five different colors. (**b**) The PCoA result demonstrates the two genetic groups (PAIO and Afrotropic). The score of the two axes (Axis 1 = 67.61%, and Axis 2 = 2.07%) (**c**) A unrooted maximum likelihood tree. All the results consistently showed the existence of the two distinct genetic lineages (PAIO and Afrotropic) for *N. albicilla* in the Indo-Pacific*.* The figure was edited by Adobe Illustrator v.22.2 (https://www.adobe.com).

We constructed a nucleotide sequence alignment set with 393 *COI* haplotypes of *N. albicilla* (Table S1), from which 186 polymorphic sites and 134 parsimony informative sites were identified. TCS network analysis (Fig. 2a) and principal coordinate analysis (PCoA) (Fig. 2b) showed the existence of two distinct genetic groups in *N. albicilla*. In the TCS network (Fig. 2a), 393 *COI* haplotypes were divided into two genetic lineages: one consisting of the 118 haplotypes detected primarily in the Afrotropic region, called the Afrotropic, and the other including 275 haplotypes found mainly in Palearctic, Australasia, Indo-Malay, and Oceania regions, called the PAIO. The lineages Afrotropic and PAIO have star-like topologies in common: NAH230 is a central haplotype in Afrotropic, and NAH03, NAH19, and NAH37 are central haplotypes in PAIO. As in the TCS network (Fig. 2a), the PCoA plot (Fig. 2b) also supported the existence of two genetic groups of *N. albicilla*. Furthermore, the TCS network (Fig. 2a) represents star-like topologies, which suggests a recent population demographic expansion for *N. albicilla.* When we calculated the pairwise *F*_ST_ values between the five different biogeographical groups of *N. albicilla*, the lowest value appeared between Palearctic and Oceania (-0.00027) and the highest between Oceania and Afrotropic (0.79859). The pairwise *F*_ST_ values between the Afrotropic and each of the other four PAIO members (0.72509–0.79859) were higher than those between the PAIO members (− 0.0027–0.01769) (Table S2). Analysis of molecular variance (AMOVA) based on *COI* were conducted to evaluate the degree of genetic differentiation statistically between PAIO and Afrotropic (Table 3). The results provide strong statistical support for the existence of two genetic lineages, PAIO and Afrotropic, with higher molecular variation between the two groups (69.88%) and lower variation within each group (8.08%). Assuming five different geographical groups, most of the variance (59.19%) was allocated to the level of individuals among the groups. The Mantel test did not show any statistically significant correlation between pairwise genetic distances and geographical distances (r = 0.166, *P* < 0.001; Fig. S2).

**Table 3:** Analysis of molecular variance (AMOVA) results performed based on the *COI* sequences of 697 *Nerita albicilla* individuals.

Groups (Lineages)	Source of variation	Degree of freedom	Sum of squares	Variance components	Percentage of variation
PAIO Afrotropic	Among groups	1	2006.590	6.86215	69.88***
	Among populations within groups	33	565.704	0.79344	8.08***
	Within populations	662	1432.357	2.16368	22.04***

Groups (Populations)	Source of variation	Degree of freedom	Sum of squares	Variance components	Percentage of variation
Palearctic Australasia Indo-Malay Oceania Afrotropic	Among groups	4	2148.831	3.94385	59.19***
	Among populations within groups	22	133.008	0.14781	2.22***
	Within populations	670	1722.813	2.57136	38.59***

Statistically significant values are indicated with asterisk: *P < 0.05, **P < 0.01, ***P < 0.001.

### Examination of species discrimination of *N. albicilla*

**Figure 3:**
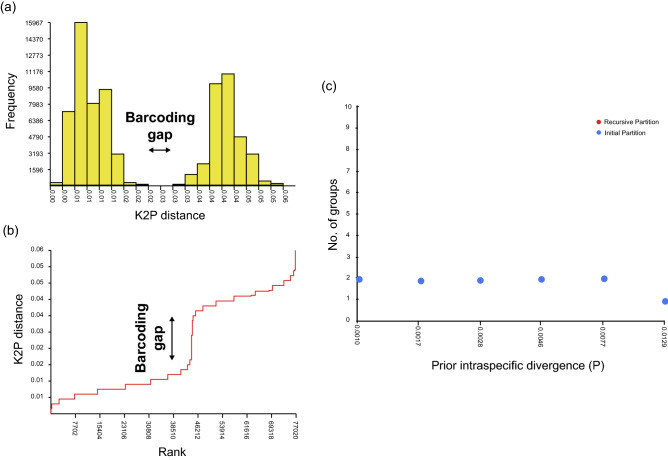
Distribution of pairwise genetic divergence, ranked pairwise difference, and automatic partition based on the 393 *COI* haplotypes of *Nerita albicilla*, indicating that it may be divided into two different species*.* (**a**) Distribution patterns of pairwise genetic divergences. The horizontal axis represents intervals of pairwise Kimura-2-parameter (K2P) genetic distance in percentage, and the vertical axis represents the number of individuals associated with each distance interval. (**b**) The result of ranked pairwise differences. The results are similar to the distribution of pairwise genetic divergence shown in (**a**). The horizontal axis indicates a ranked ordered value based on K2P genetic distance, and the vertical axis represents the K2P genetic distance in percentage. (**c**) The result of automatic partition analyses. The horizontal axis represents the prior maximum intraspecific divergence (P), and the vertical axis represents the number of groups inside the partitions (primary and recursive). The figure was edited by Adobe Illustrator v.22.2 (https://www.adobe.com).

Using Automatic Barcode Gap Discovery (ABGD), we explored the distribution of pairwise genetic divergences (Fig. 3a), ranks of pairwise genetic differences (Fig. 3b), and the results of automatic partition analyses (Fig. 3c) based on the *COI* haplotypes of *N. albicilla*, respectively. The results confirmed the existence of a distinct barcoding gap between intraspecific and interspecific variation (Fig. 3a), a distinct Kimura-2-parameter (K2P) distance gap at a specific rank (Fig. 3b), and two different groups, as shown in the automatic partition analysis (Fig. 3c). Based on the *COI* (575 bp) of *Nerita albicilla*, we identified the K2P intraspecific threshold with 393 haplotype sequences and the K2P interspecific distance ranges with 54 different species with the genus *Nerita* (Data S2, S3): NAH03 as a representative haplotype for *Nerita albicilla* (PAIO), NAH230 for *Nerita originalis* sp. nov., and the remaining 52 sequences of 52 different *Nerita* species retrieved from the GenBank database (Table S3). In Table S4, the intraspecific genetic distance ranges from 0 to 6.43% with 2.69% average (*N* = 67,575). The K2P distances between species varied from 1.80% to 26.73% (*N* = 1,431) with 16.62% mean. The pairwise K2P genetic distance between *N. albicilla* and *N. originalis* sp. nov. was 3.94%, which is higher than the mean value (= 2.69%) but lower than the maximum value (= 6.43%) in intraspecific genetic distances, whereas is lower than the mean value (= 16.62%) but higher than the minimum value (= 1.80%) in interspecific genetic distances. It may be interpreted as the two different species recently speciated (closely related with lower intraspecific distance) of *N. albicilla* and *N. originalis* sp. nov., or an identical species genetically differentiated (above the average value of intraspecific distance). Considering the existence of an apparent barcoding gap between the two species (Fig. 3), the former is likely to be more reliable than the latter. We also found the three pairwise K2P genetic distance lower than 3.94% between *Nerita* species: *N. reticulata* and *N. signata* (= 2.54%), *N. melanotragus* and *N. morio* (= 1.80%), and *N. planospira* and *N. sanguinolenta* (= 2.56%). These analyses strongly support the differentiation of *N. albicilla* into two independent species: *N. albicilla* (PAIO) and *N. originalis* sp. nov*.* (Afrotropic).

### Phylogenetic analysis and divergence time estimation

Based on the haplotype sequence alignment (Table S1), we reconstructed the maximum likelihood (ML) tree based on the 393 *COI* haplotypes with two outgroups, *N. adenensis* and *N. planospira*. The resultant ML tree revealed monophyly of the 393 *COI* haplotypes of *N. albicilla* (BP 100) and strongly supported the existence of the two distinct genetic lineages shown in population genetic analyses (Fig. S1), and strongly supported monophyly of Afrotropic (BP 100) and PAIO (BP 100). Likewise, an unrooted ML tree (without outgroups) showed two distinctive phylogenetic groups: *N. albicilla* (PAIO) and *N. originalis* sp. nov*.* (Afrotropic) (Fig. 2c). In addition, to elucidate phylogenetic relationships of *N. albicilla* and *N. originalis* sp. nov. within the genus *Nerita*, we reconstructed ML and BI trees with 133 *COI* haplotypes, which include *N. albicilla* (54 haplotypes), *N. originalis* (24 haplotypes), and 52 *Nerita* species (one haplotype per species) (Table S3). According to the well-resolved ML and BI trees (Fig. S3), *N. albicilla* (BP 100 and BPP 1.00) and *N. originalis* sp. nov. (BP 100 and BPP 0.99) formed independent monophyletic groups with high node confidence values, respectively, which are grouped together.

**Figure 4:**
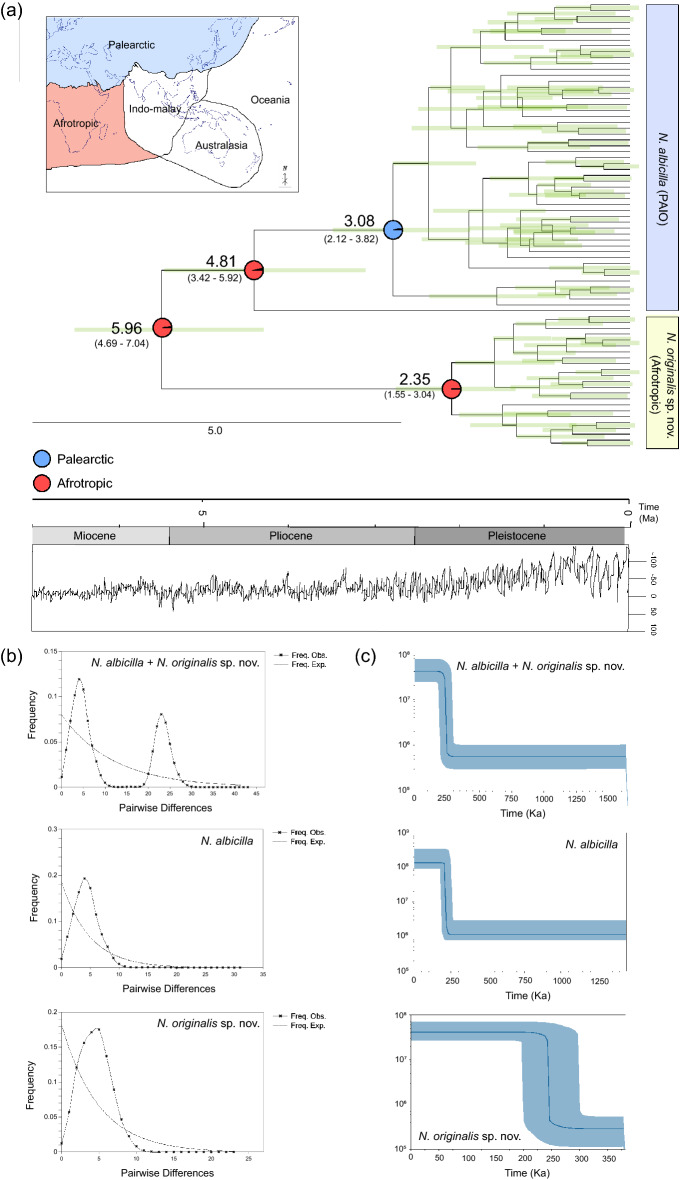
The results of time-calibrated Bayesian tree, hypothetical common ancestor using S-DIVA, mismatch distribution analyses (MDA), and Bayesian skyline plots of *Nerita albicilla* and *Nerita originalis* sp. nov. (BSPs). (**a**) Highlighted time-calibrated Bayesian tree and hypothetical common ancestor. The tree was reconstructed with 133 *COI* haplotypes, which include *N. albicilla* (54 haplotypes), *N. originalis* (24 haplotypes), and 52 *Nerita* species (one haplotype per species) (Table S3) using the BEAST (refer to Fig. S4). Additionally, a S-DIVA analysis was done with 393 *COI* haplotypes of *N. albicilla* and *N. originalis* (Table S1) to seek for the inference of ancestral areas under the Bayesian binary MCMC (BBM) model implemented in RASP ver 3.2. Ancestral areas were hypothesized based on the distribution range of the fossil records of *Nerita* and the contemporary distribution of *N. albicilla* and *N. originalis* sp. nov. Pie charts at nodes represent the probabilities of the ancestral distributions. (**b**) MDA graphs. The X-axis represents the number of pairwise differences, and the Y-axis represents the frequency of the pairwise comparisons. The observed frequencies were represented by the line marked with an x. The frequency expected under the hypothesis of the population expansion model was depicted by a continuous line. (**c**) Bayesian skyline plots. The X-axis represents time (Ka) and the Y-axis represents effective population size × mutation rate per site per generation. Medians are shown as solid lines and blue areas represent 95% HPD limits. The map provided by the d-maps site (https://d- maps.com) was edited to refer to biogeographic realms (https://ecoregions.appspot.com) by Adobe Illustrator v.22.2 (https://www.adobe.com).

As shown in Fig. 4a, the molecular clock analysis performed by using BEAST 2.6.6^19,20^ indicated that the *N. albicilla* (PAIO) and *N. originalis* sp. nov*.* (Afrotropic) shared their most recent common ancestor at about 5.96 Ma (4.69–7.04 Ma), corresponding to the late-Miocene. The *N. albicilla* lineage apparently derived from an Afrotropical ancestral haplotype (NAH324) around 4.81 Ma. In addition, dramatic diversification of *COI* haplotypes occurred mainly during the late Pliocene and Pleistocene.

### Demographic history with population expansion and dispersal

To reconstruct historical demography in terms of population expansion and dispersal, with the 393 *COI* haplotypes including 275 for *N. albicilla* (PAIO) and 118 for *N. originalis* sp. nov. (Afrotropic), we performed neutrality tests, mismatch distribution analysis (MDA), and Bayesian skyline plot (BSP) analysis. Neutrality tests (Tajima’s* D* and Fu’s *F*_S_) showed statistically significant negative values, except for the weak positive value of Tajima’s* D* observed in the TH population (Table 2). The negative values of Tajima’s *D* and Fu’s *F*_S_ tests (Table 2) provide data that support a recent expansion. The MDA results (Fig. 4b, S5a) showed that each of the five different geographical groups, as well as *N. albicilla* and *N. originalis* sp. nov. form only one unimodal curve. The unimodal characteristics also indicate that *N. albicilla* and *N. originalis* sp. nov. have undergone recent demographic expansion. However, the total that merges all the data of *N. albicilla* (PAIO) and *N. originalis* sp. nov. (Afrotropic) forms a bimodal curve, indicating the existence of two different population expansion histories. The bimodal peaks may indicate the presence of two distinct genetic lineages, representing *N. albicilla* (PAIO) and *N. originalis* sp. nov. (Afrotropic). The BSP results also showed that the effective population sizes of *N. albicilla* and *N. originalis* sp. nov. had begun to gradually increase around 250 Ka (Fig. 4c, S5b).

## Discussion

In this study, 697 *COI* sequences of *N. albicilla*, which consisted of 167 from Jeju Island, South Korea, and the remaining 530 from published data, were employed to examine the population genetic structure and taxonomy of *N. albicilla* inhabiting the Indo-Pacific region. The TCS network (Fig. 2a), PCoA (Fig. 2b), phylogenetic trees (Fig. 2c; Figs. S1 and S3), and DNA barcoding gap (Fig. 3) clearly showed that there are two different genetic lineages. The two genetic lineages were statistically supported by AMOVA (Table 3). The PAIO lineage is predominantly distributed in the Palearctic, Australasia, Indo-Malay, and Oceania regions, and the Afrotropic lineage is dominantly distributed in the Afrotropic region. This result supports the perspective of Crandall et al.^5^: Clade A corresponds to Afrotropic and Clade B to PAIO.

Our results indicate that the two genetic lineages of *N. albicilla* could be discriminated into two different species: *N. albicilla* (PAIO) and *N. originalis* sp. nov. (Afrotropic). We decided that the PAIO type continuously retains the original name of *N. albicilla* Linnaeus 1758, and the Afrotropic type is newly named *N. originalis* sp. nov.: “originalis” is a Latin word that means an English word “origin”, indicating that *N. albicilla* may have originated from *N. originalis* sp. nov. The original description of *N. albicilla* Linnaeus, 1758 was conducted with the sample from Hitoe, Tsushima, Japan which is the closest Japanese island to the Korean peninsula. Although the original description by Linnaeus^21^ was not sufficiently detailed to distinguish one from the other species, we could conclude that the PAIO type corresponds to *N. albicilla* because all individuals collected from five different Japanese sites including Okinawa I. (1), Shiova-wan(11), Cape Hedo (9), Oku (1), and Misaki (20) (Table 1) are appeared in *N. albicilla* (Clade B) without any exception (Figs. 2, S1, and S3). It implies that the Afrotropic type has never been reported from Japan so far.

The *Nerita* species, including *N. albicilla* and *N. originalis* sp. nov., display extensive dispersal potential because of their pelagic larval stage (up to 6 months)^22^. *N. albicilla* and *N. originalis* sp. nov. go through veliger larvae that remain in the plankton stage for weeks to months, floating along the flow of ocean currents in the Indo-Pacific, and are less affected by geographical barriers for dispersal. Nevertheless, in this study, it was observed that the distributional ranges of *N. albicilla* and *N. originalis* sp. nov. overlap in many locations in the Indo-Pacific, such as Jeju Island, Cook Islands, Indonesia, New Caledonia, Singapore, and South Africa, indicating broadly sympatric distributions. The sympatric distribution with their genetic differences could provide evidence for the possible species discrimination of *N. albicilla*. Through further studies including comparative morphological analyses, it may be possible to obtain additional evidence for the species discrimination of *N. albicilla* into *N. albicilla* and *N. originalis* sp. nov. The ancestral Afrotropic origin of *N. albicilla* was shown in S-DIVA and the basal-branch positioning of a South African *COI* haplotype (NAH 324) in *N. albicilla* of the ML trees (Fig. 4a; Fig. S1), which suggests that it is most likely that *N. albicilla* may have originated from *N. originalis* sp. nov. inhabiting the Afrotropic region. It is conceivable that the *N. albicilla* might have first appeared in South Africa at about 5.96 Ma and later they had spread to the Palearctic region through Indo-Malay, Australasia, and Oceania. Along the dispersal of *N. albicilla* east and north, possibly out of Africa, its *COI* haplotype diversity dramatically increased during the late Pliocene and mainly Pleistocene (ca. 5.0–1.0 Ma; Fig. 4a) with their adaptation and positive selection process to a variety of new environments^11,23^. Changes in shorelines, oceanic circulation, upwelling zones, temperatures, salinities, nutrient availability, and sea level fluctuations affect coastal habitats, causing habitat shifts in rocky habitats^24^. Interglacial transgressions may have provided opportunities for some species that survived glacial periods to expand and move into newly flooded coastal habitats^25^. During the warm periods of deglaciation, species that were able to adapt to warming conditions experienced population expansion and shifted their range northward^26^. Due to these environmental conditions, *N. albicilla* and *N. originalis* sp. nov. may have experienced dramatic population expansions. With the dispersal out of Africa and the late Pliocene and Pleistocene explosions of *COI* haplotype diversity of *N. albicilla* and *N. originalis* sp. nov., its effective population size expansions simultaneously occurred in all five regions of the Indo-Pacific area at approximately 250 Ka before the Last Glacial Maximum (LGM: ca. 0.027–0.021 Ma) (Fig. 4c, S5b).

**Figure 5:**
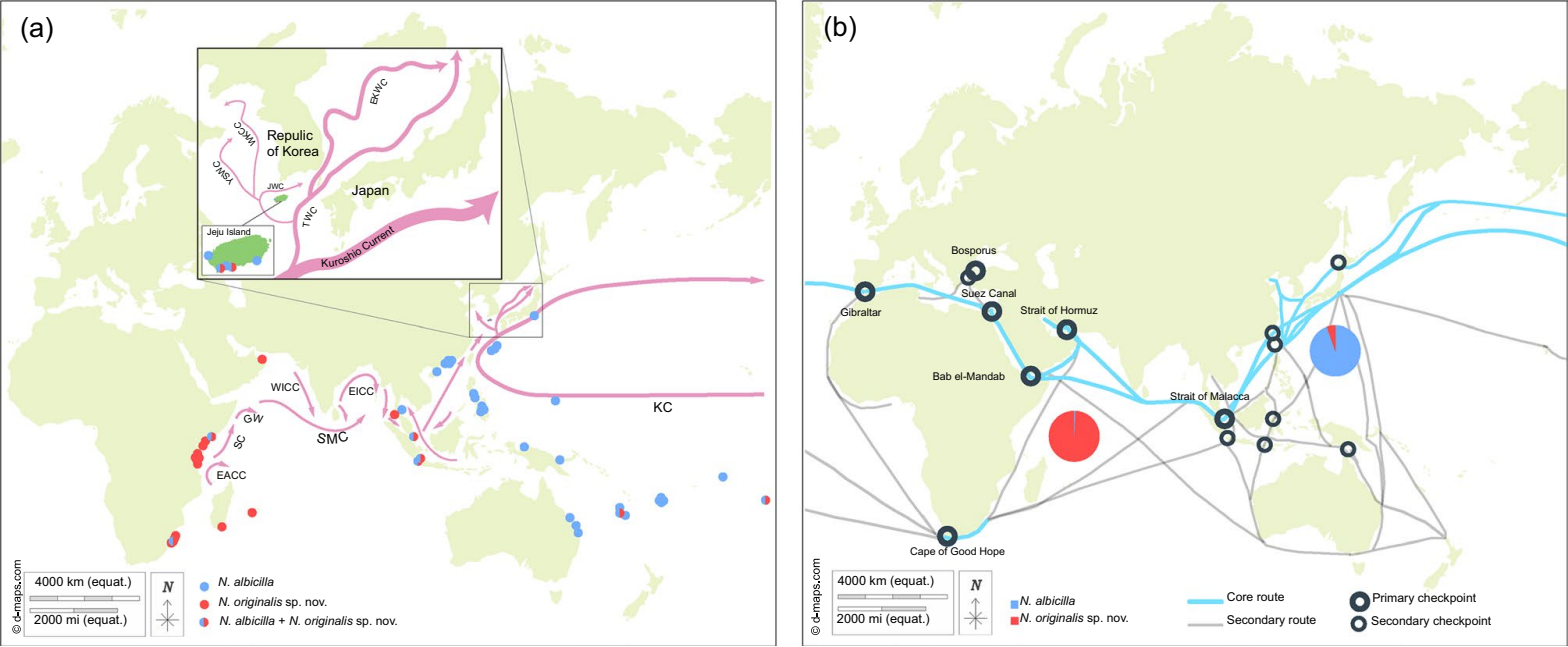
The maps of sampling sites and possible migration routes by sea currents and core and secondary maritime shipping routes that might be related to artificial migration for *Nerita albicilla* and *Nerita originalis* sp. nov. (**a**) A map of sampling sites and possible migration routes by sea currents for *Nerita albicilla* and *Nerita originalis* sp. nov. The blue dots represent the sites where only *N. albicilla* are found and the red dots represent the sites where only *N. originalis* sp. nov. are found. Mixed colored dots mean that both *N. albicilla* and *N. originalis* sp. nov. are found on the sample site. Pink lines are demonstrated as sea currents. The abbreviations of sea currents are as follows: EACC, East African Coastal Current; EICC, East India Coastal Current; EKWE, East Korean Warm Current; GW, Great Whirl; JWC, Jeju Warm Current; KC, Kuroshio Current; SC, Somali Current; SMC, Summer Monsoon Current; TWC, Tsushima Warm Current; WKCC, West Korea Coastal Current; WICC, West India Coastal Current, and YSWC, Yellow Sea Warm Current. Schematic representation of the circulation of sea current during July (summer monsoon). (**b**) A map of core and secondary maritime shipping routes in the Indo-Pacific and the pie charts representing the ratio of the number of *N. albicilla* and *N. originalis* sp. nov. In the Afrotropic region, there was only one *N. albicilla* individual and 173 *N. originalis* sp. nov. individuals. In the Palearctic, Australasia, Indo-Malay, and Oceania regions, there were 504 *N. albicilla* individuals and 19 *N. originalis* sp. nov. individuals. We found two *N. originalis* sp. nov. individuals in Jeju Island, South Korea that might have recently come north from the Afrotropic region. The basic map is from a free map providing site (https://d- maps. com) and the figure was edited refer to Soegiarto, A. & Birowo (1975) and Main Maritime Shipping Routes and Checkpoints (2011) by Adobe Illustrator v.22.2.

In general, the genus *Nerita* has a long-lived planktotrophic larval period^1,22^, which makes it possible to increase the time to dispersal, allowing a wide range of habitats to be reached^27^. The molluscan fauna of eastern Asia has been created by the repeated migration of fauna from the Indo-West Pacific Ocean^28,29^. Strong oceanic surface currents can transport planktonic larvae to distant oceanic islands, and genetic variations may accumulate, causing speciation on these islands^30–33^. Therefore, the possibility of moving to the east and north along sea currents (Fig. 5a)^34,35^ is considered the primary reason for the observed distribution. A summer monsoonal wind (southwest) can drive seasonal reversals in surface circulation patterns and water exchange, and these seasonal currents and the long planktotrophic larval period could be responsible for the larval dispersal of *N. albicilla.* Interestingly, we found two *N. originalis* sp. nov. individuals on Jeju Island, South Korea. As previously mentioned, they likely moved from the Afrotropic region through sea currents. However, recent anthropogenic introductions should not be overlooked because of the globalization of maritime trade through commercial shipping (Fig. 5b)^36–38^. Further studies are necessary to examine the major reasons and routes of their historical dispersal in Africa.

Molecular markers can be used to track the demographic responses of populations to past climatic shifts and other historical processes^39,40^, and to analyze the current intraspecific genetic variation to predict the impact of future climate change on certain populations^41^. As mentioned before, the National Institute for Biological Resources (NIBR), Ministry of Environment, South Korea selected the 100-representative organism of CBIS in 2010 which are sensitively affected by global warming inhabiting the Korean Peninsula. *N. albicilla* is included in the list of CBIS and this study is the first report with respect to this species. Through continuous monitoring of distributional changes and intraspecific genetic variations of *N. albicilla* and *N. originalis* sp. nov. could be a cornerstone for exploring and predicting the global warming effect in the Indo-Pacific with the northward migration of marine gastropods.

## Materials and methods

### Sample collection

In total, 167 *N. albicilla* individuals were collected from five intertidal regions (GJ, NJ, SC, TS, and SM) of Jeju Island on the Korean Peninsula in 2021 (Fig. 1). Collected individuals were immediately fixed with 100% alcohol. They were brought to the laboratory and stored at -20 °C. Species identification was performed using shell morphology and *COI* sequence.

### DNA extraction, PCR amplification, and sequencing

Genomic DNA was isolated from the muscle tissue (foot) using a DNeasy Blood and Tissue Kit (QIAGEN, Valencia, California, USA) following the manufacturer’s protocol. The concentration of extracted DNA was evaluated using NanoDrop 2000 (Thermo Fisher Scientific Co, USA) and 1% agarose gel electrophoresis.

To amplify partial fragments of the mitochondrial *COI* gene, PCR was performed using universal primers (LCO1490/HCO2198)^42^. The primer sequences are shown in Table S5. The thermal cycling profile consisted of denaturation at 94 °C for 2 min, 35 cycles of 94 °C for 1 min, 48 °C for 1 min, and 72 °C for 1 min; a final extension at 72 °C for 5 min; and a cooling down step was performed at 4 °C. The PCR mixtures were prepared to a total volume of 50 μl containing 1 μl of 10 pM of each primer, 1 μl of 10 mM dNTP mix, 5 μl of 10 × *Taq* DNA polymerase reaction buffer, 0.25 μl of 5 U/μl DiaSter *Taq* DNA polymerase, and 3 μl of total genomic DNA. One microliter of each PCR product was electrophoresed on a 1% agarose gel containing the eco-dye which is fluorescent dye as DNA staining solution and observed under UV light. When the PCR bands were detected, the PCR products were purified using a QIAquick PCR Purification Kit (QIAGEN Co., USA) and directly sequenced by the commercial sequencing service company SolGent Co., Ltd. (Daejeon, South Korea) with an ABI Prism 3730 DNA sequencer (PerkinElmer Inc., USA) using a Big Dye Termination Sequencing Kit (PerkinElmer Inc., USA).

### Population genetic analyses

We used *COI* sequences from 524 *N. albicilla* individuals, provided by Eric D. Crandall (https://geome-db.org/query), and six *N. albicilla* individuals which were previously reported in the NCBI GenBank database. The nucleotide sequences of mitochondrial *COI* obtained from *N. albicilla* were aligned using BioEdit 7.0.5.3^43^ and Clustal X2^44^. Pairwise genetic distances were calculated with the Kimura-2-Parameter (K2P) in Mega 11.0.13^45^. The identification of variable and parsimony informative sites and the number of haplotypes (*h*) were estimated using DnaSP 6.12.03^46^. Based on the haplotype list generated by DnaSP, the number of private haplotypes unique to each population was determined (Table S1). Descriptive statistics including the number of polymorphic sites and haplotypes, haplotype diversity, and nucleotide diversity were estimated for each population using the program DnaSP 6.12.03^46^. A haplotype network was constructed using a statistical parsimony approach at the population level using PopART^47^. To further evaluate and visualize the geographic genetic structure among the populations, PCoA was performed to visualize the geographic genetic structure among species using the DARwin 6.0.21^48^ program which ordinates genetic distance estimates calculated with the haplotype data used in this study. *F*_ST_ values between *N. albicilla* populations using *COI* data were calculated in Arlequin 3.5.2.2^49^ to test for significant differentiation among each population from sampled sites. Hierarchical analysis of molecular variance (AMOVA) was performed to examine the amount of genetic variability partitioned within and among the five groups of *N. albicilla* according to biogeographic regions (Afrotropic, Australasia, Indo-Malay, Oceania, and Palearctic) and Afrotropic and PAIO in Arlequin 3.5.2.2^49^. The correlation between pairwise genetic distances and geographic distances between individuals was established using the Mantel test (Alleles in Space 1.0^50^).

### DNA barcoding gap analyses

Analyses of barcoding gaps based on *COI* were conducted using an online version of ABGD (https://bioinfo.mnhn.fr/abi/public/abgd/abgdweb.html) to generate distance histograms, distance ranks, and automatic partitions. These analyses were conducted using the Kimura-2-parameter (K2P) distance matrix and two different parameters: the range of prior intraspecific divergence from *P*_min_ (0.001) to *P*_max_ (0.1) and relative gap width (*X* = 1.5).

### Phylogenetic analyses and divergence time estimation based on COI

Phylogenetic analyses based on the 393 *COI* haplotype sequences obtained in this study were performed using the ML method. In the ML tree, model selection in the IQ-Tree software package (http://www.iqtree.org) was tested and the substitution model HKY + F + I was chosen as the best-fit model under the Bayesian information criterion. The phylogenetic tree was analyzed from 1,000 ultrafast bootstrap replicates using the IQ-Tree web server (http://iqtree.cibiv.univie.ac.at). To elucidate phylogenetic relationships of *N. albicilla* and *N. originalis* sp. nov. within the genus *Nerita*, we additionally reconstructed ML and BI trees with 133 *COI* haplotypes (Fig. S3), which include *N. albicilla* (54 haplotypes), *N. originalis* (24 haplotypes), and 52 *Nerita* species (one haplotype per species) (Table S3). The maximum likelihood tree was reconstructed under the TIM F + I + G4 in IQtree online site and the Bayesian topology was inferred under the GTR + F + I + G4 model using MrBayes 3.2.2^51^. *Bathynerita naticoidea*, *Clypeolum owenianum*, and *Puperita pupa* were used as outgroup species.

Divergence time estimation for the two phylogenetic lineages of *N. albicilla* was conducted based on *COI* haplotype sequence matrix used in Fig. S3 by BEAST 2.6.6^19,20^. The analysis was estimated using the strict molecular clock algorithm under the calibrated-Yule model^52^, which was calibrated using the earliest fossil record data of *Nerita* (ca. 90 Ma)^53^ for the basal node (90 Ma; normal distribution) and a “monophyly” option was chosen in the BEAUti 2 program (the genus *Nerita*). Posterior distributions of the parameter were estimated using 10,000,000 MCMC iterations and sampled every 1,000 iterations after discarding the initial 20% of the iterations as burn-in. The best-fit substitution model was selected using jModelTest 2.1.7^54^, as GTR + F + I + G4. TreeAnnotator 2.6.6^55^ was used to produce a tree with maximum clade credibility and median height after removing the initial 20% of iterations as burn-in. FigTree 1.4.4^56^ was used to visualize the topology of the resultant consensus tree. To estimate the distribution of a hypothetical common ancestor, S-DIVA analysis based on the Bayesian binary MCMC method^57^ based on the BEAST tree of *COI* using the RASP 3.2 program^58^. When running the program, three possible distributed areas were coded for the ingroup taxa as follows: (A) Palearctic; (B) Afrotropic; (C) Australasia, Indo-Malay, and Oceania.

### Demographic history analyses

To investigate the demographic history of populations of *N. albicilla*, we used three approaches: neutrality tests, MDA^59^, and BSP^60^. Neutrality tests such as Tajima’s *D*^61^ and Fu’s *F*s^62^ were conducted to examine the population demographic history and evolutionary neutrality of *N. albicilla* using the Arlequin 3.5.2.2^49^ program based on *COI* haplotypes. MDA^59^ was also conducted to investigate the demographic stability of the phylogenetic lineages and species using DnaSP 6.12.03^46^. BSP^60^ was computed to examine historical demographic fluctuations since the most recent common ancestor. For the analyses, the HKY model was selected, mutation rates of 2.0 × 10^–8^ under a strict molecular clock were used with BEAST 2.6.6^19,20^, and MCMC was run for 10 million steps. TRACER 1.7.2^63^ was used to calculate the ESS value and construct the BSP.

## Supplementary Information

Supplementary Information 1.

Supplementary Information 2.

Supplementary Information 3.

Supplementary Information 4.

## Data Availability

The *COI* haplotype sequences of *Nerita albicilla* have been deposited in the GenBank database under accession numbers ON982333–ON982444 (NAH001–NAH112). The pre-processed dataset can be found in the Supplementar Data as follows: Data S1, nucleotide sequence alignment of 393 *COI* haplotypes of *N. albicilla*, Data S2, the K2P intraspecific genetic distance with 393 haplotype sequences of *Nerita albicilla,* and Data S3, the K2P interspecific distance genetic ranges with 54 different species with the genus *Nerita*.
